# Role of ADMA in the pathogenesis of microvascular complications in type 2 diabetes mellitus

**DOI:** 10.3389/fendo.2023.1183586

**Published:** 2023-04-21

**Authors:** Xinyang Guo, Yiqiao Xing, Wei Jin

**Affiliations:** Eye Center, Renmin Hospital of Wuhan University, Wuhan, Hubei, China

**Keywords:** diabetes mellitus, asymmetric dimethylarginine, microvascular complications, diabetic retinopathy, diabetic nephropathy, diabetic neuropathy, diabetic cardiomyopathy

## Abstract

Diabetic microangiopathy is a typical and severe problem in diabetics, including diabetic retinopathy, diabetic nephropathy, diabetic neuropathy, and diabetic cardiomyopathy. Patients with type 2 diabetes and diabetic microvascular complications have significantly elevated levels of Asymmetric dimethylarginine (ADMA), which is an endogenous inhibitor of nitric oxide synthase (NOS). ADMA facilitates the occurrence and progression of microvascular complications in type 2 diabetes through its effects on endothelial cell function, oxidative stress damage, inflammation, and fibrosis. This paper reviews the association between ADMA and microvascular complications of diabetes and elucidates the underlying mechanisms by which ADMA contributes to these complications. It provides a new idea and method for the prevention and treatment of microvascular complications in type 2 diabetes.

## 1 Introduction

As the global economy progresses and lifestyles change, the prevalence of diabetes is increasing annually in both developed and developing countries (1–3). According to recent statistical research, by 2040, it is anticipated that 642 million people will be diagnosed with diabetes (2). Meanwhile, there has been a significant increase in the incidence of diabetic microvascular complications (3). Diabetic microvascular complications include diabetic retinopathy, diabetic nephropathy, diabetic neuropathy, and diabetic cardiomyopathy. These complications can significantly impact patients’ quality of life and increase their risk of mortality (4, 5). The pathogenesis of diabetic microangiopathy involves several mechanisms, including activation of the protein kinase C (PKC) pathway and accumulation of advanced glycation end products (AGEs), chronic inflammation mediated by the kinin system, oxidative stress injury induced by the NADPH oxidase-reactive oxygen species (NOX-ROS) pathway, and transforming growth factor-beta (TGF-β)-induced fibrosis (6).

Previous studies have suggested that asymmetric dimethylarginine (ADMA), an endogenous inhibitor of nitric oxide synthase, plays an important role in the pathogenesis of diabetic microangiopathy (7). AGEs can enhance ADMA synthesis, leading to endothelial dysfunction (8). Furthermore, ADMA is positively correlated with C-reactive protein (CRP) (9), and can activate macrophages and monocytes to mediate inflammatory responses (10, 11). Additionally, ADMA may promote tissue fibrosis by upregulating TGF-β expression (12). However, the precise mechanisms underlying the effects of ADMA in diabetic microvascular complications remain poorly understood. The aim of this literature review is to provide an overview of the role of ADMA in the pathogenesis of microvascular complications in diabetes and to suggest potential therapeutic strategies and novel therapeutic targets for the prevention and treatment of these complications.

## 2 Synthesis and metabolism of ADMA

### 2.1 Synthesis of ADMA

**Figure 1:**
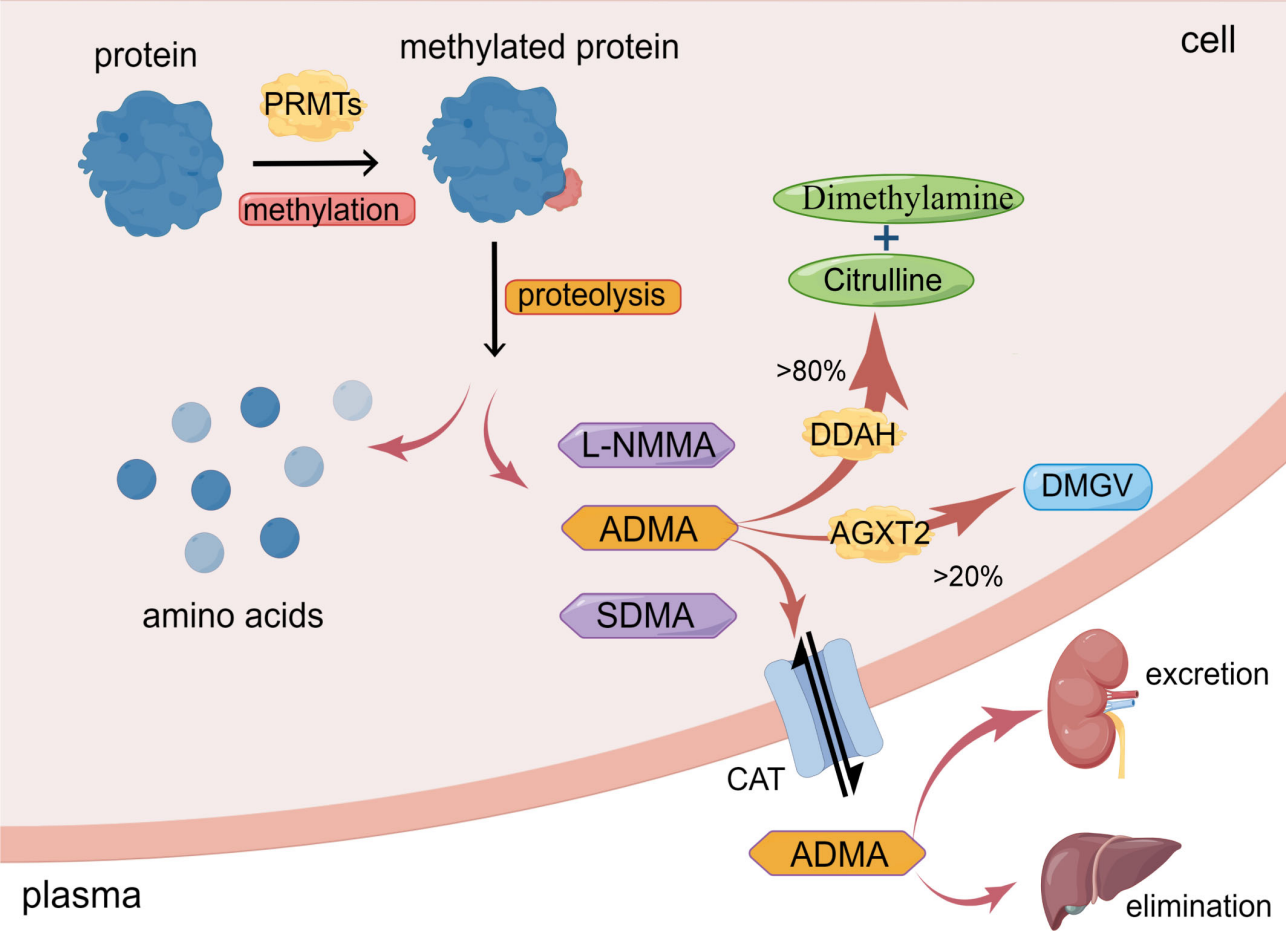
Synthesis and metabolism of ADMA. ADMA is formed by the post-transcriptional methylation of proteins mediated by PRMTs. Amino acids and methylarginines such as ADMA, L-NMMA, and SDMA are subsequently released during protein proteolysis. >80% ADMA is hydrolyzed by DDAH to dimethylamine and citrulline; <20% ADMA is degraded by AGXT2 to DMGV. ADMA can also enter circulation via CAT and excretion by the kidney or elimination by the liver. (The figure was created using Figdraw).

Asymmetric dimethylarginine (ADMA) is generated from post-translational methylation modification of arginine residues in proteins by protein arginine methyltransferases (PRMTs) and is released during proteolysis (13). ADMA is widely present in mammalian bodies, including plasma, tissue fluids, and cytoplasm. There are three known arginine residues, namely NG-monomethyl-L-arginine (L-NMMA), asymmetric NG, NG-dimethyl-L-arginine (ADMA), and symmetric NG, N’G-dimethyl-L-arginine (SDMA) (14, 15). Type I protein arginine methyltransferases (PRMT-I) or type II protein arginine methyltransferase (PRMT-II) can catalyze the formation of L-NMMA, whereas PRMT-I and PRMT-II catalyze the formation of ADMA and SDMA, respectively (16, 17). As endogenous nitric oxide synthase (NOS) inhibitors, L-NMMA and ADMA play an essential role in inhibiting nitric oxide (NO) production (14). However, while SDMA does not significantly affect NOS expression, it still inhibits NO synthesis, most likely due to its ability to competitively inhibit arginine transport (18). Interestingly, the concentration of ADMA in the blood is significantly higher than that of NMMA (15). As a result, over the past few decades, a growing number of studies have focused on the effects of ADMA on vascular diseases, while NMMA and SDMA have received less attention (19, 20) (**Figure 1**)

Under normal physiological conditions, healthy adults have an average plasma ADMA level ranging between 0.4-0.6μmol/L (21). However, in pathological conditions such as hypertension and hypercholesterolemia, the plasma level of ADMA in patients can increase up to two times (21). In patients with chronic renal failure, the plasma level of ADMA can increase by almost eight times (22). This increase in ADMA levels in pathological conditions may be due to the increased synthesis and decreased degradation or excretion of ADMA. Physiologically, the intracellular concentration of ADMA is around 3.6 μmol/L, resulting in a mere 10% reduction in the production of nitric oxide (NO). However, if the plasma level of ADMA is increased by 3-9 times, it can significantly impact the intracellular concentration of ADMA, leading to a much greater inhibitory effect on NO synthesis, ranging from 30% to 70% (23, 24).

Intracellular L-arginine can produce L-citrulline and NO under the catalysis of NOS (25). ADMA competes with the substrate L-arginine for binding to the active site of NOS, leading to a reduction in NO synthesis (26). Nevertheless, this inhibitory effect can be reversed by the exogenous addition of L-arginine (22). This suggests that maintaining an appropriate ratio of L-arginine to ADMA may be crucial for proper NOS function. High levels of ADMA can lead to reduced production of NO, which impairs endothelial function and increases the risk of cardiovascular events such as atherosclerosis and hypertension (27). However, L-arginine therapy has been found to be effective in improving endothelium-mediated vasodilation in individuals with elevated ADMA levels (28). Apart from its role in mediating endothelial dysfunction, ADMA also has the potential to induce oxidative stress by promoting the uncoupling of NOS (29). Elevated ADMA levels inhibit NOS activity and promote NOS uncoupling, resulting in the generation of ROS and peroxynitrite ions (ONOO-) that mediate intracellular oxidative stress and ultimately lead to cell damage (30).

Moreover, recent research suggests that ADMA may have NOS-independent functions in microangiopathy. Increased levels of ADMA promote inflammation and fibrosis in endothelial cells, which could potentially contribute to the onset and advancement of microangiopathy. Research has demonstrated that ADMA activates the nuclear factor kappa B (NF-κB), p38 mitogen-activated protein kinase (p38 MAPK), and extracellular signal-regulated kinase (ERK) pathways in endothelial cells, leading to the secretion of tumor necrosis factor-alpha (TNF-α) and soluble intercellular adhesion molecule-1 (sICAM-1) (31). These molecules mediate the inflammatory response of endothelial cells. ADMA can also induce tissue fibrosis by promoting Epithelial-to-Mesenchymal Transition (EMT) or Endothelial-to-Mesenchymal Transition (EndMT) (12). These effects were found to be unrelated to ADMA’s inhibitory effect on NOS.

### 2.2 Metabolism of ADMA

The metabolism of ADMA includes three pathways. Firstly, ADMA is mainly hydrolyzed by dimethylarginine dimethylaminohydrolase (DDAH), which specifically metabolizes ADMA and NMMA and does not hydrolyze SDMA (12, 14, 32). Intracellular ADMA is mainly hydrolyzed by DDAH to citrulline and dimethylamine (12, 14). Leiper et al. originally found in 1999 that two isoforms of DDAH exist in mammals, DDAH1 and DDAH2 (33). DDAH1 is predominantly expressed in tissues that express neuronal nitric oxide synthase (nNOS), such as the brain tissue, whereas DDAH2 is mainly expressed in tissues that express endothelial NOS (eNOS) and inducible NOS (iNOS), such as vascular endothelium and immune tissue (34).

Secondly, a minor portion of ADMA is decomposed to α-keto-δ-(NG, NG-dimethylguanidino) valeric acid (DMGV) by alanine-glyoxylate aminotransferase 2 (AGXT2) (12, 35). Despite the limited role of AGXT2 in clearing ADMA, there are still studies showing reduced NO synthesis and increased blood pressure in AGXT2 knockout mice (36).

Thirdly, ADMA exits cells into blood circulation through the cationic amino acid transporter family (CAT) and is excreted primarily through the kidneys or eliminated in the liver (12, 35, 37). Meanwhile, circulating ADMA also could enter cells via CAT to exert its biological effects (12, 35). Under conditions of inflammation, oxidative stress, and hyperglycemia, the activity of DDAH is inhibited (35), resulting in a reduction in the degradation of ADMA. ADMA deposition in the cells eventually leads to cellular dysfunction (**Figure 1**).

## 3 ADMA and diabetic microangiopathy

**Figure 2:**
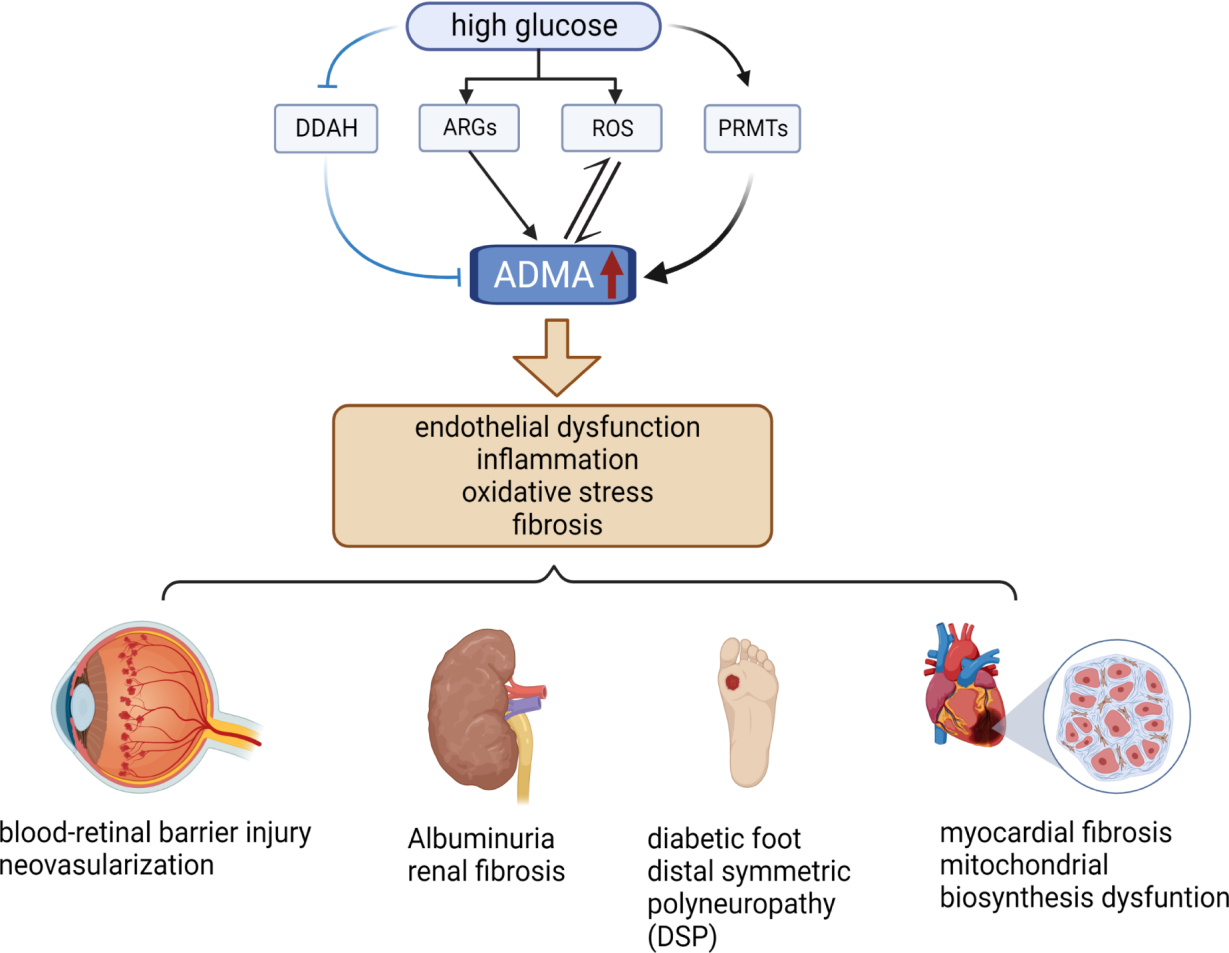
Relationship between ADMA and diabetic microvascular complications. High levels of glucose increase the level of ARGs, ROS, and PRMTs while decreasing the level of DDAH, resulting in elevated levels of ADMA. Elevated levels of ADMA can stimulate even more ROS production and further increase ADMA levels. Elevated ADMA induces endothelial dysfunction, inflammation, oxidative stress, and fibrosis. These pathological changes cause several diabetic microvascular complications, such as DR, DN, DF, DSP, and DCM. (The figure was created with BioRender.com).

ADMA serves as a biomarker for endothelial cell dysfunction and is involved in a diverse range of pathological processes, including inflammation, angiogenesis, tissue fibrosis, and oxidative stress (12, 20, 38) (**Figure 2**). Notably, ADMA has been established as a potent and independent prognostic marker of several cardiovascular diseases and chronic kidney disease (27, 39). Meanwhile, ADMA is also closely associated with diabetic microvascular complications. ADMA has been shown to be a more specific predictor of diabetic microvascular complications compared to other markers such as glycated hemoglobin and N-ε-(carboxymethyl)lysine (CML) (40). Higher plasma ADMA concentrations are strongly associated with the development of diabetic microvascular complication (41). Additionally, the duration of diabetes is positively correlated with plasma ADMA concentrations, and hence the longer the duration of diabetes, the higher the risk of microvascular complications (42, 43). Overall, these results indicated that ADMA is an important biomarker for warning of retinal microvascular injury in diabetes mellitus.

### 3.1 ADMA and diabetic retinopathy

As people’s living standards improve and their dietary habits change, the incidence of diabetes and its associated complications has increased significantly. Among these complications, diabetic retinopathy (DR) is the most prevalent microvascular complication and a leading cause of visual impairment in the working-age population (44, 45). DR can be classified into non-proliferative diabetic retinopathy (NPDR) and proliferative diabetic retinopathy (PDR) based on the severity of retinal lesions. NPDR typically occurs in the early stages of the disease and is primarily characterized by augmented vascular permeability, which leads to microaneurysms, hemorrhage, hard exudates, and cotton wool-like spots (46, 47). PDR typically manifests in the late stages of DR and is characterized by fibrovascular proliferation, resulting in neovascularization, vitreous hemorrhage, and traction retinal detachmentt (46, 47).

ADMA, as a risk factor for microvascular injury, has been linked to the development and progression of DR. The association between ADMA and DR was initially established in 2007 by Malecki et al., who reported that increased circulating levels of ADMA were linked to DR and that ADMA was an independent predictor of the disease (48). This finding was later supported by Abhary, S. et al., who also detected significantly elevated serum ADMA levels in patients with severe DR (49). Additionally, Motohiko et al. reported significantly higher levels of ADMA in the serum and aqueous humor of diabetes patients with DR compared to those without diabetes mellitus (50). ADMA is synthesized by PRMT1, and PRMT1 gene polymorphisms have been shown to be associated with an increased incidence of proliferative DR (PDR), further highlighting the critical role of ADMA in PDR (51). Elevated levels of ADMA have been found not only in severe DR but also in prediabetic and diabetic stages, indicating that ADMA may play an essential role in both the development and progression of DR (52). Given the significant correlation between plasma ADMA levels and the development of DR, researchers have investigated the relationship between plasma ADMA levels and retinal imaging. Dag et al. reported that plasma ADMA levels were elevated and choroidal thickness was reduced in patients with PDR compared to the NPDR group and controls, indicating a potential link between ADMA and retinal imaging (53). However, Hernández et al. did not find any correlation between ADMA plasma levels and retinal neurological dysfunction and structural alterations (54). Therefore, further studies are needed to fully elucidate the relationship between ADMA and diabetic retinopathy imaging, as well as to explore its potential correlation with retinal neurological dysfunction and structural changes.

Metabolomics has gained widespread attention from researchers in recent years due to its ability to explore disease pathogenesis and potential therapeutic targets through the qualitative and quantitative analysis of small molecule metabolites in an organism. Researchers have conducted metabolomics studies on DR patients using various biological samples such as serum, plasma, vitreous humor, atrial fluid, and cerebrospinal fluid (55). Jun Ho Yun et al. utilized a targeted metabolomics approach to analyze serum samples from non-DR, NPDR, and PDR patients and identified several differential metabolites (56). They found that total methylarginine (DMA) concentrations were significantly higher in DR patients than in non-DR patients (56). Furthermore, they observed that PDR patients had higher total DMA concentrations compared to NPDR patients (56). The study revealed that the severity of DR was positively correlated with DMA levels, highlighting the possible role of DMA in DR progression. Moreover, in a study by Huiyi Jin et al., the metabolism of aqueous humor from patients with DR was analyzed using a 1H-NMR-based metabolomics approach (57). The researchers found that DMA levels in aqueous humor were significantly higher in patients with DR than in diabetic patients without DR, providing further evidence that DMA may serve as a potential biomarker for DR (57). Total DMA included ADMA and SDMA (56). Compared with SDMA, ADMA plays a more important role in inhibiting the activity of NOS and inducing endothelial cell injury (58). Additionally, researches have shown that the arginine and proline metabolic pathways are also associated with DR patients (25, 55, 59, 60). Since ADMA is generated by the methylation of arginine residues, elevated levels of arginine metabolic pathways may affect the concentrations of ADMA. This increase in ADMA levels can inhibit the activity of NOS, leading to a reduction in NO production, and promote NOS uncoupling, which can produce superoxide anions and lead to oxidative stress injury (55, 61). Therefore, the dysregulation of arginine and proline metabolic pathways may play a role in the pathogenesis of DR by affecting ADMA levels and NO production. Further research is needed to fully understand the relationship between these metabolic pathways and DR, and to determine if they can be targeted for therapeutic intervention in DR patients.

Recent studies have explored the mechanisms of microvascular injury in DR associated with ADMA. ADMA affects NO synthesis, resulting in hemodynamic disorders and ROS production, which causes oxidative stress in endothelial cells (62). Oxidative stress in endothelial cells is a critical contributor to the development of retinal microvascular injury in patients with diabetic retinopathy (DR). Specifically, in a high glucose environment, DDAH expression is down-regulated, while PRMT expression is up-regulated, leading to an increase in the synthesis of asymmetric dimethylarginine (ADMA) (63–65) (**Figure 2**). Elevated levels of ADMA can further exacerbate oxidative stress-mediated retinal endothelial cell damage by increasing the production of ROS (64). (**Figure 2**) Additionally, Huang et al. demonstrated that ADMA can damage the blood-retinal barrier by affecting the expression of blood-retinal barrier-specific component connexin 43 (Cx43) from diabetic rats induced by streptozotocin (STZ) (66). The disruption of the blood-retinal barrier is responsible for the formation of microaneurysms, hemorrhage, and hard exudates, which are characteristic of NPDR. Moreover, Du et al. reported that ADMA can induce neovascularization by promoting the proliferation, migration, adhesion, and tube formation of choroid-retinal endothelial cells (RF/6A) (67). Neovascularization is a hallmark of PDR and a critical cause of vitreous hemorrhage, which can lead to severe visual impairment. The studies mentioned above indicate that reducing the level of ADMA may provide protective benefits to the blood-retinal barrier in patients with diabetes and can potentially decrease the formation of neovascularization, thereby delaying the progression of DR. Many studies have shown that DDAH1 is more closely related to the metabolism of ADMA (32, 68, 69). However, the expression of DDAH2 in endothelial cells is higher than that of DDAH1, and DDAH2 co-localizes with endothelial nitric oxide synthase (eNOS) (70, 71). In the oxygen-induced retinopathy mouse model (OIR), both DDAH1 and DDAH2 are expressed in retinal tissues, but DDAH2 expression was significantly higher in retinal than in brain, indicating that DDAH2 has higher specificity in retinal (72). Therefore, increasing the expression of DDAH2 or enhancing its activity may alleviate ADMA-mediated retinal microvascular endothelial cell injury.

### 3.2 ADMA and diabetic nephropathy

Diabetic nephropathy (DN) is a major complication of diabetes and a leading cause of severe renal function impairment in diabetic patients. It is characterized by the presence of albuminuria and progressive glomerulosclerosis, which can eventually lead to renal failure (73, 74). DN is also a primary cause of the end-stage renal disease (ESRD) (73). The pathogenesis of DN is complex and involves several factors, including hemodynamic changes, oxidative stress, renal fibrosis, and inflammation (75). One critical player in the pathogenesis of DN is ADMA, an inhibitor of NOS. ADMA plays a crucial role not only in regulating hemodynamics, but also in inducing oxidative stress injury, promoting fibrosis, and mediating inflammation (9, 12).

Several studies have demonstrated the close correlation between ADMA and DN. A study by Tanhäuserová et al. found that ADMA is negatively correlated with renal function, such as glomerular filtration rate (GFR), and that the ADMA/GFR ratio is an essential biomarker for predicting the progression of DN (76). In patients with advanced chronic kidney disease (CKD), the higher the ADMA level, the greater the reduction in GFR and the more severe the kidney injury (77). Furthermore, for patients who progressed to ERSD and required hemodialysis treatment, serum levels of ADMA were significantly higher in DN patients than in non-DN patients both before and after dialysis (78). It suggested that ADMA has higher specificity in DN compared to other CKD. Among haemodialysis patients, the level of plasma ADMA is a powerful and independent predictor for both cardiovascular events and overall mortality (79). This highlights the potential of ADMA as a useful biomarker for both the diagnosis and monitoring of DN.

Albuminuria, as a prominent clinical feature of DN, is closely related to ADMA. A prospective study conducted in Brazil revealed that patients with elevated plasma ADMA levels had a higher risk of developing albuminuria in both the hypertension group and the hypertension-diabetes group (80). In addition, the odds ratio of ADMA in the logistic regression analysis was higher in the hypertension-diabetes group than in the hypertension group (80), suggesting that the presence of diabetes exacerbates the risk of ADMA-induced albuminuria. Furthermore, Yilmaz et al. observed a positive correlation between serum level of ADMA and proteinuria and a negatively correlated with endothelial cell function (81). ADMA may promote the progression of albuminuria by damaging renal microvascular endothelial cells. Meanwhile, Kaida et al. have found that proteinuria can induce oxidative stress injury, which further up-regulated the expression of PRMT1 and increase the synthesis of ADMA in renal tubular cells (82). The interaction between ADMA and proteinuria can worsen renal function. Reducing the synthesis or promoting the breakdown of ADMA may slow down the progression of proteinuria. Therefore, ADMA may be a therapeutic target for alleviating albuminuria in patients with DN, and its specific mechanism remains to be further verified.

DDAH is a key enzyme in ADMA degradation. Wetzel et al. have found that overexpression of DDAH1 in mice can reverse ADMA-mediated albuminuria, oxidative stress injury, and inflammatory response, thus improving DN (83). Meanwhile, reduced expression of DDAH, significant accumulation of ADMA in plasma and renal tissue, tubular necrosis, and significantly impaired renal function was found in ischemia-reperfusion-injured mice (84). However, in folate nephropathy and unilateral ureteric obstruction models, renal tissue damage and renal fibrosis were less severe in DDAH1 knockout mice than in controls (85). Whether DDAH inhibition has a protective effect on renal function in DN patients remains to be further verified.

Interstitial fibrosis and glomerulosclerosis are major causes of chronic renal failure in DN patients. ADMA, as a mediator of renal tissue fibrosis, plays an essential role in this process. ADMA has been found to promote the deposition of collagen fibers and damage capillaries of the glomerulus and tubule, thereby aggravating renal fibrosis (12, 86). The underlying mechanism involves ADMA up-regulating hypoxia-inducing factor (HIF) and its downstream target molecule, endothelin-1(ET-1), as well as promoting the expression of Transforming Growth Factor β (TGF-β) (87, 88). This leads to the transdifferentiation of fibroblasts, epithelial cells, or endothelial cells into myofibroblasts, ultimately leading to increased synthesis of extracellular matrix and progression of chronic kidney disease (89–91). Furthermore, Isaivani et al. demonstrated that ADMA can activate the fibrotic signaling pathway through the NOX4/ROS/ERK pathway, leading to increased synthesis of extracellular matrix and accelerating kidney cell fibrosis (92). In rats induced by monocrotaline, DDAH1 knockdown significantly exacerbated oxidative stress, pulmonary vascular remodeling, and lung fibrosis (93). Through the same mechanism, elevated ADMA may mediate oxidative stress and fibrosis of renal tubular epithelial cells and glomerular microvascular endothelial cells, eventually leading to renal failure. Therefore, the reduction of ADMA levels may be a therapeutic strategy for delaying the progression of CKD induced by renal fibrosis.

### 3.3 ADMA and diabetic neuropathy

Diabetic neuropathy is a common complication of diabetes, seriously affecting patients’ quality of life. The most common type of diabetic neuropathy is distal symmetric polyneuropathy (DSP), which manifests as sensory abnormalities in a stocking-glove distribution in the limbs, including numbness, pain, and weakness (94). Severe DSP can progress to diabetic foot, which may ultimately lead to limb amputation (95). High blood sugar plays a critical role in the development of diabetic neuropathy by inducing oxidative stress and inflammation. However, maintaining glycemic control alone is insufficient to halt the progression of neuropathy (94), suggesting the involvement of other crucial molecules.

As mentioned previously, ADMA is a critical mediator of oxidative stress damage and inflammation, and its role in exacerbating diabetic neuropathy remains controversial. Several studies have found that patients with type 2 diabetes who suffer from peripheral neuropathy exhibit endothelial dysfunction (94–96). ADMA can induce endothelial dysfunction by affecting NO synthesis and promoting oxidative stress. However, the use of antioxidants, such as alpha-lipoic acid (ALA), can improve ADMA-induced endothelial damage and promote the repair of peripheral sensory nerve function (97). Stojanovic et al. found that compared with the control group, plasma ADMA levels were significantly increased in patients with type 2 diabetes mellitus complicated with DSP (98). ADMA can be used as a marker to detect the progression of diabetic neuropathy. Diabetic foot (DF) is an important manifestation of diabetic peripheral neuropathy in patients with diabetes. Hala et al. found that the level of circulating ADMA was significantly higher in DM patients who had DF compared to those without DF, and NO levels were significantly lower in DM patients with DF (99). ADMA may affect the microcirculation of the limbs by affecting the synthesis of NO, resulting in aseptic ulcers in the feet.

However, Kyrillos et al. suggested that the plasma levels of ADMA were not significantly increased in patients with DF compared with the control group (100). Moreover, Halit et al. found no significant difference in ADMA levels between diabetic patients with neuropathy and without neuropathy (101). The differences in these results may be attributed to inadequate sample size or individual differences in the baseline disease of patients. Further clinical controlled studies with larger samples are necessary to confirm the relationship between ADMA and diabetic neuropathy.

### 3.4 ADMA and diabetic cardiomyopathy

Diabetic cardiomyopathy (DCM) refers to an abnormality in the structure and function of the myocardium that occurs without other traditional cardiovascular risks, such as coronary artery disease, valvular heart disease, hypertension, or hyperlipidemia (102). The early manifestation of DCM includes impaired diastolic function, which may progress to systolic dysfunction and eventually lead to heart failure (103). Although it is commonly believed that DCM is closely associated with diabetic macroangiopathy, studies as early as the 1980s have found evidence of capillary basement membrane thickening and microangioma formation within the myocardium of diabetic patients (104–106). These findings suggest that myocardial microangiopathy also plays a significant role in the development and progression of diabetic cardiomyopathy.

The pathogenesis of DCM involves various factors, including hyperglycemia, insulin resistance, hyperlipidemia, oxidative stress, inflammatory responses, activation of the renin-angiotensin-aldosterone system, and abnormal function of the sympathetic nervous system (107). Studies have shown that abnormal glucose metabolism can lead to oxidative stress, reducing the availability of nitric oxide (NO) and synthesis of vascular endothelial growth factor (VEGF), causing dysfunction of myocardial microvascular endothelial cells, and promoting vasoconstriction, ultimately leading to reduced blood flow to myocardial cells (108, 109). In addition, hyperglycemia increases the plasma level of ET-1 and promotes the endothelial-to-mesenchymal transformation (EndMT) of myocardial microvascular endothelial cells (110). This process can further trigger myocardial fibrosis, which plays a significant role in ventricular remodeling and the development of myocardial systolic and diastolic dysfunction.

In diabetic rat models with DDAH2 overexpression, a decrease in left ventricular end-diastolic pressure and an increase in systolic pressure were observed, along with an improvement in myocardial function (111). In addition, DDAH2 administration to cardiomyocytes cultured in a high-glucose environment resulted in a reduction in ADMA synthesis, an increase in NOS levels, and a decrease in type I collagen fiber, matrix metalloproteinase 2 (MMP2), and tissue inhibitor of metalloproteinase 2 (TIMP2) levels (111). MMP2 and TIMP2 are crucial factors in the synthesis of myocardial extracellular matrix (ECM) and are implicated in the progression of myocardial fibrosis (112). Reducing ADMA levels may alleviate myocardial microvascular injury, inhibit myocardial fibrosis, improve myocardial remodeling, and enhance myocardial function.

In a rat model of diabetic cardiomyopathy, it was observed that ADMA levels were elevated in cardiomyocytes, which interfered with mitochondrial biosynthesis and affected myocardium function (113, 114). The underlying mechanism could be that ADMA up-regulated the expression of coupled protein 2 (UCP2) and inhibited the activity of the peroxisome proliferator-activated receptor-γ-coactivator-1α (PGC1α) promoter, leading to the down-regulation of PGC-1α expression and, thus affecting ATP synthesis (113, 114). However, resveratrol treatment helped to mitigate ADMA accumulation and reverse Adma-mediated PGC-1α expression reduction and acetylation, which ultimately improve myocardial mitochondrial function (115). The findings indicate that managing ADMA levels could be an effective strategy in the treatment of diabetic cardiomyopathy, and resveratrol might have the potential as a therapeutic agent.

## 4 Discussion

The above findings suggest that ADMA is an important marker of microvascular injury in diabetic patients and is involved in the development and progression of diabetic microvascular complications through multiple pathways. Endothelial dysfunction, inflammation, oxidative stress damage, and fibrosis in endothelial cells are important risk factors for diabetic microvascular complications. Many studies in the past have focused on the effects of ADMA on endothelial cell function through the inhibition of NOS activity and NO production. In recent years, more and more studies have confirmed the important role of ADMA in inducing oxidative stress injury and fibrosis, which brings a new direction for the treatment of diabetic microvascular complications. More studies are still needed in the future to further analyze the mechanistic pathways involved in ADMA, to find key therapeutic targets, and to provide new ideas for the prevention and treatment of diabetic microvascular complications.

ADMA is a by-product of protein arginine methylation and can be significantly increased in various pathological conditions like diabetes, hypertension, coronary heart disease, and chronic kidney disease. This elevation may be caused by an imbalance between ADMA production and degradation. Although ADMA can be partially eliminated through urine excretion, the main pathway for ADMA metabolism is intracellular DDAH degradation, which highlights the importance of PRMTs and DDAH in maintaining normal ADMA levels. Further exploration is required to determine the mechanism by which the expression of these two enzymes can be affected in pathological conditions. Additionally, it has been suggested that the occurrence of PDR could be linked to polymorphisms in the PRMT1 gene (51). However, it is currently unknown whether similar associations exist in other patients with diabetic microvascular complications, as well as whether there is concurrent DDAH gene polymorphism. Further research is required to address these questions.

Elevated ADMA levels have been linked to adverse effects, prompting researchers to explore methods for reducing them. One such approach involves L-arginine, which has been shown to reverse the inhibitory effect of ADMA on NOS (22). However, while L-arginine has been shown to improve endothelial function in individuals with high ADMA levels (28), some studies have produced unexpected results. These studies have found that L-arginine supplementation may not have any positive effects on blood vessels and can even lead to adverse outcomes (23). Moreover, higher L-arginine levels correlate with increased ADMA levels (23). This phenomenon may be attributed to the competitive inhibition of DDAH enzyme activity by higher L-arginine levels, which subsequently reduces ADMA degradation (23). Therefore, the role of arginine in improving endothelial dysfunction remains controversial. Further research is needed to fully understand the potential benefits and adverse effects of L-arginine supplementation for endothelial function and vascular health. Additionally, while studies indicate that various medications like antihypertensive, lipid-lowering, hypoglycemic, and antioxidant drugs can reduce ADMA levels, most of these studies lack placebo controls (116). Currently, no specific drug targets ADMA levels. Therefore, there is a need for further investigation into ADMA-lowering medications, and additional clinical studies are required to determine whether reducing ADMA levels can lead to a significant slowdown of diabetic microvascular complications progression and better prognoses.

## Author contributions

WJ contributed to conception and design of the review. XG wrote the first draft of the manuscript and draw the figures. WJ and YX contributed to manuscript revision, read, and approved the submitted version.
